# Electro-acupuncture for irritable bowel syndrome with constipation: study protocol of a pilot, randomized, double-blinded, sham-controlled trial

**DOI:** 10.3389/fneur.2025.1632822

**Published:** 2025-09-12

**Authors:** Wai Ching Lam, Huangyan Chen, Kewin Tien Ho Siah, Elyse R. Thakur, Linda L. D. Zhong

**Affiliations:** ^1^Biomedical Sciences and Chinese Medicine, School of Biological Sciences, Nanyang Technological University, Singapore, Singapore; ^2^Department of Epidemiology, Harvard T.H. Chan School of Public Health, Boston, MA, United States; ^3^Division of Gastroenterology and Hepatology, University Medicine Cluster, National University Hospital, Singapore, Singapore; ^4^Department of Medicine, Yong Loo Lin School of Medicine, National University of Singapore, Singapore, Singapore; ^5^Division of Gastroenterology and Hepatology, Atrium Health, Charlotte, NS, United States; ^6^Section of Gastroenterology, Wake Forest University School of Medicine, Winston-Salem, NS, United States

**Keywords:** acupuncture, irritable bowel syndrome, constipation, randomized controlled trial, food and drug administration

## Abstract

**Introduction:**

Irritable bowel syndrome with constipation (IBS-C) is a common functional gastrointestinal disorder characterized by constipation, abdominal discomfort, and a substantial impact on patients’ quality of life. While acupuncture could be effective in treating general IBS, high-quality clinical evidence specifically supporting its efficacy in IBS-C remains limited. Our study aims to address this gap by evaluating the effectiveness and safety of electro-acupuncture in managing IBS-C symptoms.

**Methods:**

This a multicentre, randomized, double-blinded, sham-controlled trial. A total of 60 IBS-C patients are randomized to receive either electro-acupuncture (n = 30) or sham acupuncture (n = 30). Patients undergo a 2-week screening period, followed by 6 weeks of treatment (12 sessions) and 6 weeks of follow-up. From the beginning of the screening period to the end of the trial, patients are instructed to complete daily diaries recording bowel movement timing, stool consistency, straining severity, sensation of complete or incomplete evacuation, and the name and dosage of any medications taken. The primary outcome measure follows the FDA-recommended endpoint for IBS-C trials. Additionally, biological samples are collected to explore the potential mechanisms underlying the effects of acupuncture on IBS-C pathology.

**Ethics and dissemination:**

The protocol (version 1.4.4) has received approval from the Institutional Review Board of Nanyang Technological University (IRB-2023-451).

**Trial registration number:**

NCT06219707.

## 1 Introduction

Affecting approximately 10% of the general population, irritable bowel syndrome (IBS) is one of the most common functional gastrointestinal disorders in clinical practice (1). As a subtype of IBS, studies suggested IBS with constipation (IBS-C) accounts for one-third of total IBS patients under the Rome IV criteria (2, 3).

As a disorder of gut-brain interaction, IBS-C patients continuously suffer from symptoms such as abdominal pain, bloating, and infrequent, hard, or lumpy stools. Furthermore, patients frequently suffer from psychological distress and co-morbidities such as anxiety and depression (4). These symptoms not only reduce patients’ quality of life, but also pose a significant healthcare burden due to frequent medical visits (5). Despite the availability of pharmacological and dietary interventions, many IBS-C patients experience recurrent symptoms and dissatisfied treatment effects, highlighting the need for additional therapeutic options (6).

Recent research suggests that alterations in gut microbiota composition, neurotransmitter metabolism, and short-chain fatty acid (SCFA) production may play a crucial role in the pathophysiology of IBS-C (7–10). The gut-brain axis, a bidirectional communication pathway between the central nervous system and the gastrointestinal tract, is thought to be dysregulated in IBS-C patients, contributing to abnormal gut motility, visceral hypersensitivity, and psychological comorbidities (10–13).

Given these multifactorial mechanisms, there is growing interest in exploring non-pharmacological interventions, such as acupuncture, to modulate the brain-gut axis and restore gut homeostasis (14). Evidence suggests that acupuncture may influence gut motility, reduce visceral hypersensitivity, and regulate neurotransmitter-related pathways, leading to the alleviation of both gastrointestinal and psychological symptoms in IBS-C patients (15, 16).

Several clinical trials have shown that acupuncture can alleviate global symptoms and improve quality of life (17, 18). However, acupuncture studies focusing on IBS-C are limited by methodological shortcomings, including insufficient IBS-C-specific outcomes, inadequate blinding protocols, and a lack of sham-controlled designs (19, 20). Consequently, the therapeutic effects of acupuncture on IBS-C, particularly its impact on gut microbiota composition and brain-gut interactions, are not well understood.

In this acupuncture trial, we aim to evaluate the efficacy and feasibility of electro-acupuncture (EA) for IBS-C. We hypothesize that EA would result in superior symptom improvement compared to sham acupuncture. Additionally, through the collection of biological samples during the trial, we are going to investigate changes in the gut microbiota composition and related metabolites to explore potential mechanistic links between EA interventions and clinical outcomes.

## 2 Methods and analysis

### 2.1 Study design

**Figure 1:**
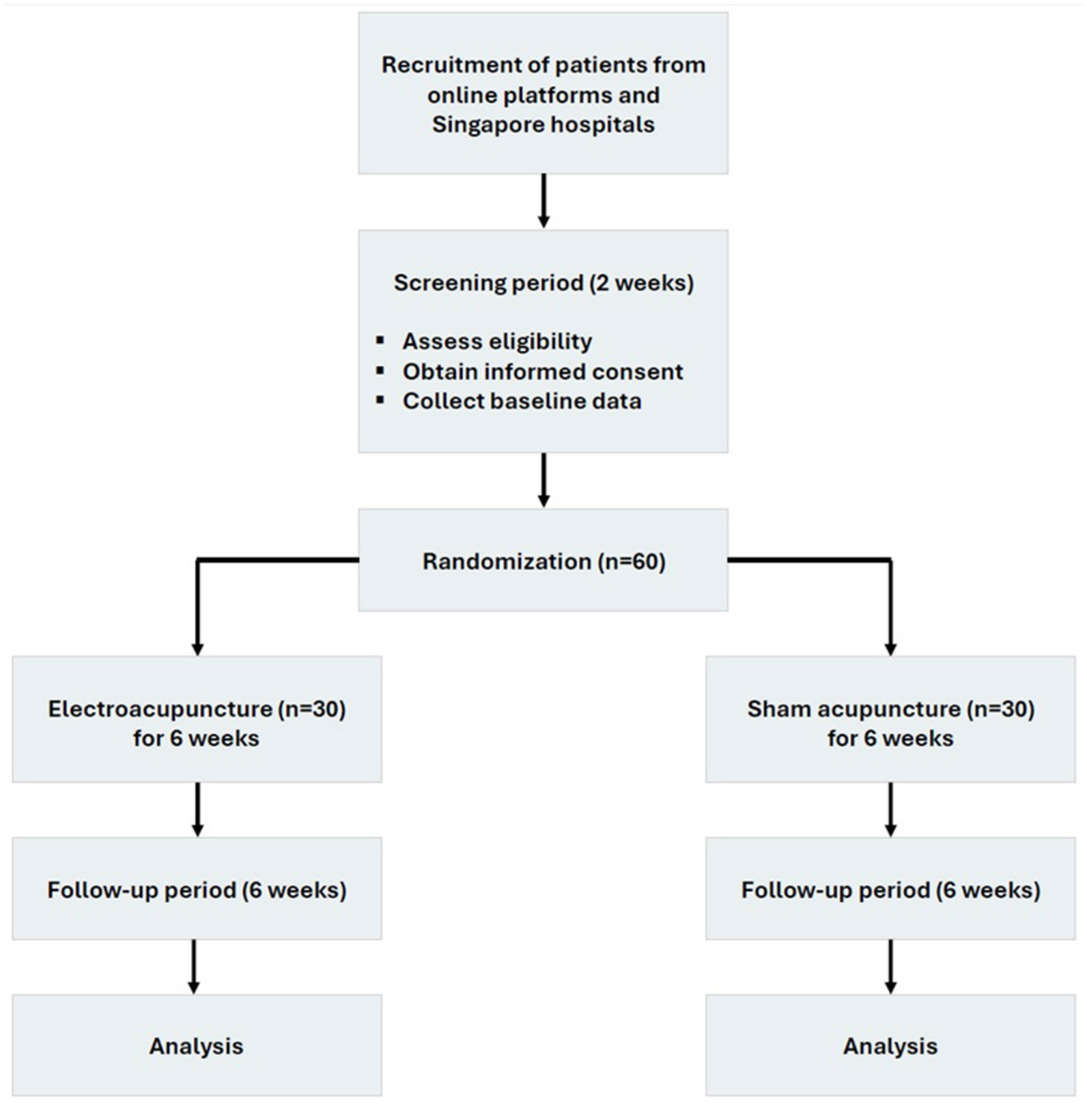
Study flow.

This pilot, randomized, double-blinded, sham-controlled trial is being conducted in Singapore since September 2024 (with the first patient enrolled). The study design strictly adheres to the principles of Declaration of Helsinki, Good Clinical Practice (GCP) guidelines, and mirrors STandards for Reporting Interventions in Clinical Trials of Acupuncture (STRICTA) guideline and the Consolidated Standards of Reporting Trials (CONSORT) statement, including the CONSORT-Outcomes 2022 extension (21, 22). The study lasts for a total of 14 weeks. All eligible patients first undergo a 2-week screening period, which includes providing blood samples for routine testing prior to enrollment. After the screening period, eligible patients are randomized in a 1:1 ratio to receive either EA or sham acupuncture over a 6-week intervention period, followed by a 6-week follow-up phase (Figure 1; Table 1). Sham acupuncture is selected as to offset the placebo effect of acupuncture, and there is no standard medication in treating IBS-C.

**Table 1:** Schedule of study procedures.

Week	0	2	3	4	5	6	7	13
Visit	1	2–3	4–5	6–7	8–9	10–11	12–14	15
Eligibility assessment	✓	✓						
Informed consent	✓							
Baseline data collection	✓							
Concomitant drug record	✓	✓	✓	✓	✓	✓	✓	✓
Adverse effects assessment		✓	✓	✓	✓	✓	✓	✓
Physical examination and samples collection	✓	✓					✓	
Food frequency questionnaire (FFQ)	✓							
Short-form Wang Qi’s body constitution classification questionnaire (SF-CCMQ)	✓			✓			✓	✓
Questionnaires with psychiatric assessment	✓			✓			✓	✓
Intervention		✓	✓	✓	✓	✓	✓	

Patients are required to fill in their diaries daily from the start of the 2-week screening period until the end of the trial, recording the timing of bowel movements (BMs), stool consistency, degree of straining during defecation, sensation of complete or incomplete evacuation, and the name and dosage of any medications taken. Throughout the trial, patients are asked to avoid making significant lifestyle changes, such as initiating a new diet or modifying their exercise routine. Patients must discontinue any medications listed in the exclusion criteria before entering the trial. Concomitant medications that may affect IBS symptoms, such as antibiotics, antidepressants, probiotics, or herbal medicine, are prohibited for at least 1 month before the screening period. If a participant meets the inclusion criteria but needs to use medications due to a comorbid illness or changes in health status, it will be recorded in the case report form (CRF).

### 2.2 Eligibility criteria and recruitment

Participants are included in the study if they meet the following inclusion criteria: (1) Fulfilment of the Rome IV criteria for IBS-C (23); (2) Age of 21 to 65 years (inclusive); (3) Weekly average of worst daily abdominal pain score of ≥3 (0–10 scale) for at least 12 weeks before the first visit and during screening period (24); (4) < 3 complete spontaneous bowel movements (CSBMs) per week for at least 12 weeks before the first visit and during screening period (24); (5) Written informed consent. Exclusion criteria include (1) Pregnancy or breast-feeding; (2) Medical history of inflammatory bowel diseases, carbohydrate malabsorption, hormonal disorder, known allergies to food additives, and/or any other serious diseases; (3) History of gastrointestinal tract segment removal or bariatric surgery for obesity; (4) Appendectomy or cholecystectomy within the past 2 months, or other abdominal surgeries within the past 6 months prior to trial enrollment; (5) Unstable medical conditions that could be associated with abdominal pain or discomfort and could potentially influence the assessments in this trial (e.g., chronic kidney disease, endometriosis, lactose intolerance); (6) Diagnosed with primary severe mental illness; (7) Patients who have received acupuncture treatment in last 3 months, or took concomitant medication with affect gastrointestinal motility or visceral sensation, such as antidiarrheal agent, antidepressant, narcotic analgesic, and anticholinergic; (8) Alcoholism or drug abuse in past 1 year; (9) Having needle phobia or allergy to acupuncture needle materials; (10) Antibiotics and probiotics/prebiotics usage in the previous month; (11) Participating in other clinical studies.

Participants are recruited from online platforms and Singapore hospitals [National University Hospital (NUH), NTU Lee Kong Chian School of Medicine (NTU LKC Medicine), Singapore General Hospital (SGH), and Tan Tock Seng Hospital (TTSH)]. Acupuncture interventions are conducted at the Nanyang Technological University (NTU) Chinese Medicine clinic, as well as at a collaborative Traditional Chinese Medicine (TCM) clinic at Kwong Wai Shiu (KWS) Hospital. This collaborative clinic, which has received standardized training provided by the NTU research team, is restricted from collecting or handling outcome data from patients except for safety-related purposes. Subject recruitment advertisements approved by the NTU Institutional Review Board (IRB) are placed on the social network platform and in the hospitals. Participants who are interested in the trial can contact the investigators by telephone, e-mail, or online registration. According to the inclusion and exclusion criteria, a standardized screening form is used to identify the potential eligible participants. Once a potential eligible subject is confirmed, an investigator communicates remotely with the subject, explaining the purpose, procedures, nature of the study, to arrange dates for the subsequent in-person meetings. All participants are informed that they are free to withdraw from the study at any time.

### 2.3 Intervention and control

**Figure 2:**
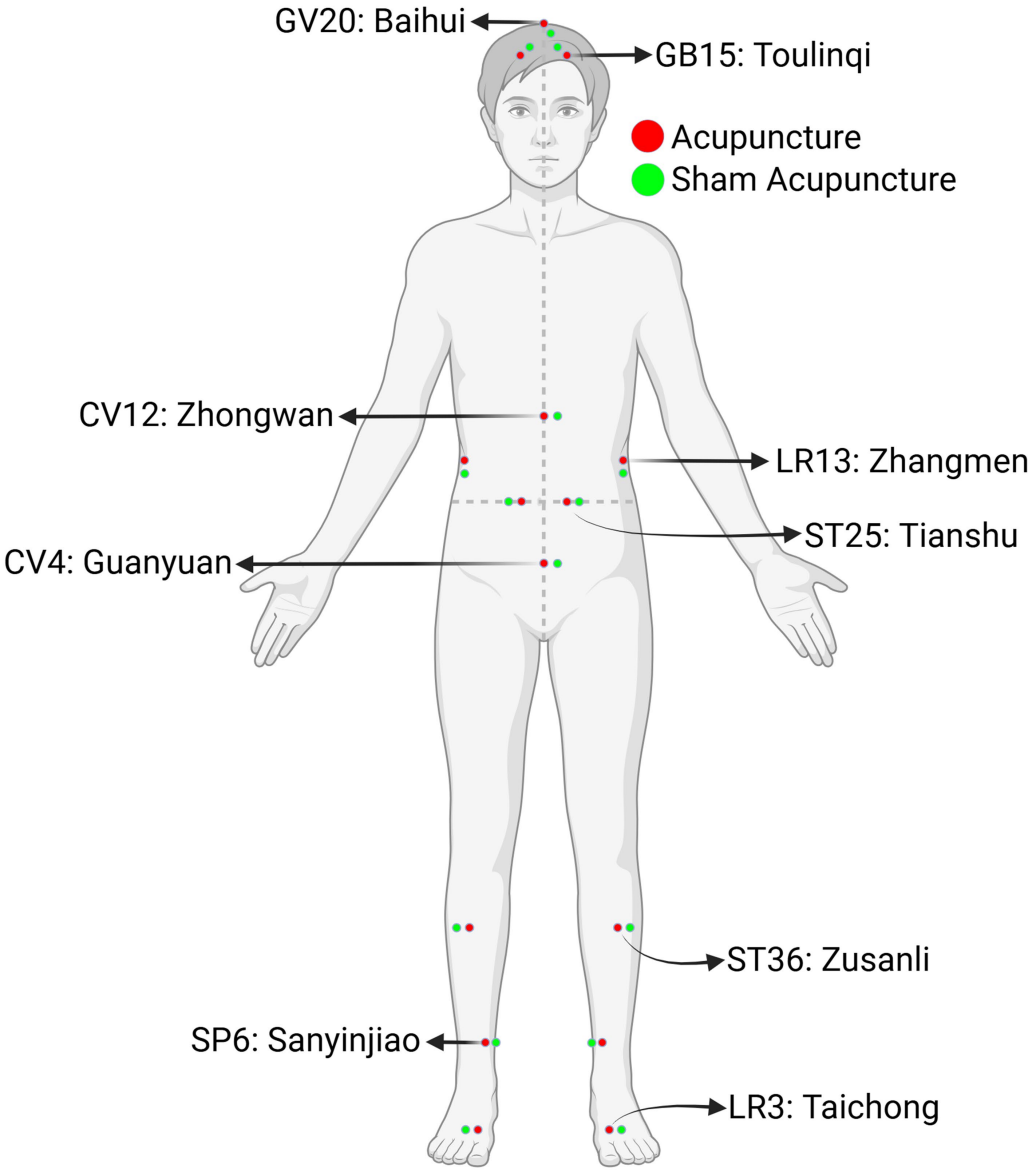
Anatomical locations of acupuncture and sham points.

Each patient is scheduled for a total of 12 treatment sessions, 30 min for each session, 2 times a week over a 6-week period. The selection of acupuncture points is based on TCM theory and evidence-based clinical research (18). According to the TCM theory, the Spleen transforms the food digested by the Stomach into essences and helps to transport throughout the body, while the Liver ensures the smooth flow of Qi, modifies the activity of internal organs (Zang Fu), and is highly related to the emotional and psychological state. Therefore, most TCM experts agree that the weakness of the Spleen and Stomach is the basic pathogenesis of IBS. In IBS-C patients, the syndrome of dryness and heat in the intestines indicates a further imbalance in fluid metabolism (25). The principles of treatment for IBS-C are to regulate the Qi movement among the Liver and Spleen, soften stools, and promote BMs. The following eight acupuncture points are selected for treatment: Baihui (GV20), Toulinqi (GB15), Taichong (LR3), Zhangmen (LR13), Sanyinjiao (SP6), Zhongwan (CV12), Guanyuan (CV4), Tianshu (ST25), Zusanli (ST36) (Table 2; Figure 2).

**Table 2:** Depths and anatomical locations of acupuncture and sham points used in the study.

Acupoint/Sham-Acupoint	Depth	Location
GV20: Baihui	0.5–1 cun	On the head, 5 cun superior to the anterior hairline, on the anterior median line.
Sham Baihui		1 cun 22.5 degrees lateral (left side of patient) to the midline and anterior of Baihui.
GB15: Toulinqi	0.3–0.5 cun	On the head, 0.5 cun within the anterior hairline, directly superior to the centre of the pupil.
Sham Toulinqi		1 cun 45 degrees superior and medial of Toulinqi.
CV12: Zhongwan	0.8–1.5 cun	On the upper abdomen, 4 cun superior to the centre of the umbilicus, on the anterior median line.
Sham Zhongwan		1 cun lateral (left side of patient) of Zhongwan.
CV4: Guanyuan	0.8–1.5 cun	On the lower abdomen, 3 cun inferior to the centre of the umbilicus, on the anterior median line.
Sham Guanyuan		1 cun lateral (left side of patient) of Guanyuan.
ST25: Tianshu	1–1.5 cun	On the upper abdomen, 2 cun lateral to the centre of the umbilicus.
Sham Tianshu		1 cun lateral of Tianshu.
LR13: Zhangmen	0.5–1 cun	On the lateral abdomen, inferior to the free extremity of the 11th rib.
Sham Zhangmen		1 cun to the inferior of Zhangmen.
ST36: Zusanli	1–1.5 cun	On the anterior aspect of the leg, on the line connecting ST35 with ST41, 3 cun inferior to ST35.
Sham Zusanli		1 cun lateral of Zusanli.
SP6: Sanyinjiao	1–1.5 cun	On the tibial aspect of the leg, posterior to the medial border of the tibia, 3 cun superior to the prominence of the medial malleolus.
Sham Sanyinjiao		1 cun posterior of Sanyinjiao.
LR3: Taichong	0.5–0.8 cun	On the dorsum of the foot, between the first and second metatarsal bones, in the depression distal to the junction of the bases of the two bones, over the dorsalis pedis artery.
Sham Taichong		1 cun to the lateral of Taichong between the second and third metatarsal bones.

For the EA group, disposable acupuncture needles (0.30 mm in diameter and 25–40 mm in length) are inserted obliquely at a depth of 10–30 mm into scalp acupuncture points (Baihui, Toulinqi) or straightly into body acupuncture points (Taichong, Zhangmen, Sanyinjiao, Zhongwan, Guanyuan, Tianshu, Zusanli). EA is applied to the abdominal points using fast and dispersed waves through an electric needle stimulator (ES-160 6-channel electro-acupuncture device) for 30 min. The waveform frequency is set to 5 Hz, and the current intensity is adjusted based on the patient’s comfort level, with licensed TCM practitioners continuously monitoring and adjusting the intensity during treatment to ensure safety and efficacy. The alternating stimulation is believed to produce maximal biochemical responses in the brain (26).

For the sham acupuncture group, disposable acupuncture needles (0.30 mm in diameter and 25–40 mm in length) are inserted to the similar depth as in the EA group, but at sham acupuncture points (Sham-Baihui, Sham-Toulinqi, Sham-Taichong, Sham-Zhangmen, Sham-Sanyinjiao, Sham-Zhongwan, Sham-Guanyuan, Sham-Tianshu, Sham-Zusanli; Table 2, Figure 2). The sham acupuncture points are located 1 cun away from the actual acupuncture points. For acupuncture points along the midline, the sham acupuncture points are positioned 1 cun to the left of the actual acupuncture points (based on the patient’s perspective). The sham points are not acupuncture points nor located on meridians (27). Both the EA and sham acupuncture groups follow the same procedures, including connection to the stimulator device for the same duration. In the sham group, although no electrical current is delivered, the power switch is turned on and the knobs are manipulated to produce audible clicking sounds, mimicking auditory cues of active treatment.

### 2.4 Outcomes and data collection

Daily symptoms recorded in physical or online patient diary include the number of BMs, SBMs, CSBMs, along with the severity of abdominal pain, discomfort, straining, and bloating, assessed using an 11-point scale (0 = none, 10 = worst possible). For each BM, stool consistency (7-point Bristol Stool Form Scale), sensation of severe straining during defecation, and use of medication are also recorded. Data will be entered electronically and stored in an encoded database within 24 h of receipt, with double data entry and range checks. Access to the trial dataset is restricted to investigators.

The primary outcome of the study is defined as responders who have a decrease in the weekly average of worst abdominal pain scores of ≥30% compared with baseline and a weekly increase of ≥1 CSBM from baseline (24). To assess the response over time, data between 2 groups will be summarized and compared throughout the treatment period and at the end of follow-up.

Other measurements include: (1) Proportion of patients with an improvement of ≥30% in abdominal pain scores from baseline; (2) Proportion of patients with an average increase of 1 or more CSBMs per week as compared with the baseline; (3) Average number of SBMs, CSBMs per week; (4) Percentage of bowel movements with normal consistency; (5) Percentage of bowel movements with severe or very severe straining during defecation; (6) Median time to the first SBMs, CSBMs after first session of EA; (7) Average number of bisacodyl tablets or enemas used per week; (8) Global assessment of efficacy of treatment with Likert scale at mid-treatment, end of the treatment, and end of follow-up; (9) IBS-C related severity and quality of life scores such as IBS Symptom Severity Scale (IBS-SSS), IBS Quality of Life (IBS-QOL), Patient Assessment of Constipation Symptoms (PAC-SYM), Constipation Quality of Life (PAC-QOL) and Patient Health Questionnaire-15 (PHQ-15) at the start of the treatment, mid-treatment, end of the treatment, and end of follow-up; (10) Depression, anxiety, and stress symptoms in IBS-C patients by Hamilton Depression Scale (HAMD), Self-rating Depression Scale (SDS), Patient Health Questionnaire (PHQ-9), General Anxiety Disorder (GAD-7), Hospital Anxiety and Depression Scale (HADS), Visceral Sensitivity Index (VSI) and Perceived Stress Scale (PSS) at the start of treatment, end of treatment, and end of follow-up; (11) Patient expectancy and trust in treatment, assessed via a brief questionnaire at the start of treatment, end of treatment, and end of follow-up; (12) Feasibility outcomes, including recruitment and retention rates, adherence to treatment protocols, completeness and quality of symptom diaries and questionnaire responses, and documentation of adverse events throughout the study. In addition to the primary and secondary outcomes, dietary intake and TCM constitutional patterns are assessed using the Food Frequency Questionnaire (FFQ) and Short-Form Wang Qi’s Body Constitution Classification Questionnaire (SF-CCMQ).

To facilitate participant compliance, several measures are implemented throughout the study. First, an informed consent process is conducted with all participants, providing information on the study schedule, potential side effects of treatment, participant responsibilities, and confidentiality, along with additional consent for collection and use of participant biological specimens (Supplementary File). Second, a 2-week screening period is conducted to identify and exclude ineligible or potentially non-compliant participants before randomization. Third, participants receive weekly reminders for upcoming appointments and the completion of their diaries. If a participant considers withdrawing from the study, the study team will engage with them to understand the reasons and explore possible solutions to address their concerns to retain the participant in the study.

### 2.5 Sample size calculation

Our study is the first sham-controlled acupuncture trial that uses the Food and Drug Administration (FDA) recommended end point for IBS-C. The sample size was determined based on the study’s objectives, statistical precision, feasibility, and previously reported clinical response rates in IBS-C acupuncture trials. We referenced a randomized controlled trial by Pei et al. (28), which reported a significant difference in clinical effective rates between the acupuncture and control group. Assuming a two-sided alpha of 0.05, power of 80%, an expected response rate of approximately 70% in the acupuncture group versus 30% in the sham control group, and accounting for a 20% dropout rate and potential missing data, a total sample size of 60 patients (30 in the experimental arm and 30 in the control arm) was designed to detect clinically meaningful differences.

### 2.6 Randomization, blinding and concealment

Simple, complete, non-sequential random numbers were generated in advance by a computer program in blocks of four by a statistician from a third-party institution and were kept by the principal investigator. After a participant’s eligibility is confirmed, a randomization number which corresponds to the group allocation is provided to the acupuncturist. This arrangement ensures that both the clinical assessor and participants are blinded to the allocation. To strengthen the blinding effect, eye masks are used to cover the participants’ eyes during the treatment.

The trial uses patient-blind technique, which means needles for EA and sham acupuncture are of identical looking and use same package. The study remains blinded until the final statistical analysis and the preparation of the final report. In the event that emergency code-breaking is required, the principal investigator will be informed. Investigators who break the code must document the reasons in the patients’ notes. Emergency code-breaking may occur under specific circumstances, including but not limited to (1): a serious adverse event (SAE) that is considered to be related to the interventions; or (2) a serious complication arises.

### 2.7 Safety assessments

Data from patients who have had at least one acupuncture or sham-acupuncture session are included in the assessment of acupuncture safety. Safety profiles are assessed by documenting important AEs reported during each treatment session and in follow-up interviews. The severity and causal relationship of AEs to the interventions are assessed by the investigators. Additionally, physical examinations and clinical laboratory tests are conducted to monitor patient safety.

### 2.8 Samples collection and healthy control recruitment

For the analysis of fecal microbiota and related metabolites, blood, urine, and stool samples are collected before and after treatment and will be analyzed under standardized laboratory testing process. In addition to the IBS-C participants, 30 healthy volunteers aged 21 to 65 years (inclusive) are recruited from the general population through flyers and online platforms. These individuals must not have any diagnosed gastrointestinal disorders, chronic diseases, or recent use of antibiotics or probiotics. The healthy control group serves as a baseline reference for microbiota composition and metabolite levels, helping to distinguish disease-specific features in IBS-C patients and access changes induced by EA. These volunteers do not receive any interventions and are included only for observational comparison.

### 2.9 Statistical analyses

All efficacy and safety analyses will be conducted based on intention-to-treat (ITT) principle. Missing values will be imputed by the last-observation-carried-forward method. Statistical analysis will be performed using the Stata software, with statistical significance defined as a two-sided *p*-value of <0.05. Baseline characteristics will be reported as mean (SD). Baseline differences between the groups will be evaluated using Student’s t-test for normally distributed continuous variables and the non-parametric Mann–Whitney U test for non-normally distributed variables. For categorical variables, the chi-squared test or Fisher’s exact test will be applied. Comparisons between groups will be conducted by using the unpaired t-test for normally distributed data and the Mann–Whitney test for non-normally distributed data. Within-group differences will be evaluated using the paired t-test for normally distributed data and the Wilcoxon signed-rank test for non-normally distributed data.

To minimize the risk of false positives, multiple-testing correction methods will be applied during the data analysis process where appropriate. Additionally, advanced statistical methods, including the Logistic Generalized Linear Mixed Model (GLMM), will be applied. Potential inter-center differences in study outcomes will also be assessed.

### 2.10 Ethics and dissemination

The trial is designed and conducted in accordance with the ethical principles outlined in the Declaration of Helsinki and the GCP guidelines. The protocol, informed consent forms, recruitment materials, and all participant materials have received approval from the NTU IRB (IRB-2023-451). Approval of both the protocol and the consent form have been obtained before any participant is enrolled. Any amendment to the protocol will require review and approval by the IRB before the changes are implemented.

### 2.11 Dissemination of findings

The entire study is estimated to be completed within 3 years. De-identified results will be published in peer-reviewed scientific journals and reports to organizations, including the NTU IRB. Study findings will also be shared through social media and academic events.

### 2.12 Patient and public involvement

Patients or members of the public were not directly involved in setting the research question, designing the study, selecting outcome measures, or contributing to recruitment. Patients will be informed about the study results upon completion.

## 3 Discussion

This randomized, double-blinded, sham-controlled pilot trial is designed to rigorously evaluate the efficacy and safety of EA in the management of IBS-C. To our knowledge, this is the first sham-controlled acupuncture study for IBS-C. Additionally, it is the first acupuncture trial to adopt the FDA-recommended primary endpoint for IBS-C. By incorporating a biological sampling strategy to explore mechanistic insights, our comprehensive approach enables assessment not only of clinical outcomes but also of how EA may modulate gut microbiota and neurotransmitter-related metabolites associated with the brain-gut axis.

The double-blind design ensures that neither the participants nor the accessors are aware of the treatment allocation, minimizing potential biases in outcome assessment. In addition to the use of eye masks, identical acupuncture needles are applied for both the EA and sham groups. While participants may experience different sensations from the electrical stimulation, they are informed in advance that these sensations could vary from person to person. These approaches help strengthening the blinding effect between the groups.

It is important to acknowledge that the insertion of needles in the sham group may still induce minor physiological effects (29, 30). The purpose of using sham acupuncture in this study is not to compare the effects of needle insertion versus non-insertion, but to assess the difference between acupuncture at acupoints versus non-acupoints based on TCM theory. Sham acupuncture, which involves needle insertion outside these designated acupoints, serves as a necessary control measure to ensure blinding of the study. By comparing the physiological responses between the two groups, we can explore whether acupuncture at traditional acupoints provides distinct effects compared to non-acupoint areas. This approach allows us to isolate the potential impact of acupuncture grounded in traditional meridian theory, rather than just the mechanical effects of needle insertion.

Moreover, we recognize the importance of excluding patients with potential organic diseases. In addition to obtaining colonoscopy reports where available, we have implemented a comprehensive screening process. This includes blood tests, medical consultations, patient diaries, and questionnaires covering personal and family gastrointestinal history, as well as any recent unexplained weight loss. These factors are carefully considered when determining participants’ eligibility.

This study has several limitations. First, as a pilot trial with 60 participants, the findings should be interpreted with caution. Further studies with larger sample sizes are needed to confirm the results. Second, as a pilot trial conducted in Singapore, the limited geographic diversity may affect the external validity of the results. Cultural factors, diet, and genetic backgrounds could influence both the response to acupuncture and the baseline microbiota composition. Third, while sham acupuncture controls for expectation and placebo effects, there remains the possibility of physiological responses due to needle insertion. Lastly, the 6-week follow-up period may be insufficient to fully capture the long-term sustainability of EA’s effects.

In conclusion, this trial is expected to generate high-quality evidence on the clinical efficacy and potential mechanisms of electro-acupuncture for IBS-C. The findings could support EA as a non-pharmacological treatment option, inform future large-scale trials, and provide new insights into the gut-brain-microbiota interface in functional gastrointestinal disorders.
